# *Pax6* regulates the formation of the habenular nuclei by controlling the temporospatial expression of *Shh* in the diencephalon in vertebrates

**DOI:** 10.1186/1741-7007-12-13

**Published:** 2014-02-14

**Authors:** Mallika Chatterjee, Qiuxia Guo, Sabrina Weber, Steffen Scholpp, James YH Li

**Affiliations:** 1 Department of Genetics and Developmental Biology, University of Connecticut Health Center, 400 Farmington Avenue, Farmington, CT, 06030-6403, USA; 2 Karlsruhe Institute of Technology (KIT), Institute of Toxicology and Genetics, 76021 Karlsruhe, Germany; 3 Current address: Department of Biological Sciences, Tata Institute of Fundamental Research, 1 Homi Bhabha Road, Colaba, Navy Nagar, Mumbai 400005, India

**Keywords:** Organizer activity, Signaling, Forebrain, Habenula, Thalamus, Mice, Zebrafish

## Abstract

**Background:**

The habenula and the thalamus are two critical nodes in the forebrain circuitry and they connect the midbrain and the cerebral cortex in vertebrates. The habenula is derived from the epithalamus and rests dorsally to the thalamus. Both epithalamus and thalamus arise from a single diencephalon segment called prosomere (p)2. Shh is expressed in the ventral midline of the neural tube and in the mid-diencephalic organizer (MDO) at the zona limitans intrathalamica between thalamus and prethalamus. Acting as a morphogen, Shh plays an important role in regulating cell proliferation and survival in the diencephalon and thalamic patterning. The molecular regulation of the MDO *Shh* expression and the potential role of Shh in development of the habenula remain largely unclear.

**Results:**

We show that deleting paired-box and homeobox-containing gene *Pax6* results in precocious and expanded expression of *Shh* in the prospective MDO in fish and mice, whereas gain-of-function of *pax6* inhibits MDO *shh* expression in fish. Using gene expression and genetic fate mapping, we have characterized the expression of molecular markers that demarcate the progenitors and precursors of habenular neurons. We show that the thalamic domain is shifted dorsally and the epithalamus is missing in the alar plate of p2 in the *Pax6* mutant mouse. Conversely, the epithalamus is expanded ventrally at the expense of the thalamus in mouse embryos with reduced Shh activity. Significantly, attenuating Shh signaling largely rescues the patterning of p2 and restores the epithalamus in *Pax6* mouse mutants, suggesting that *Shh* acts downstream of *Pax6* in controlling the formation of the habenula. Similar to that found in the mouse, we show that *pax6* controls the formation of the epithalamus mostly via the regulation of MDO *shh* expression in zebrafish.

**Conclusions:**

Our findings demonstrate that Pax6 has an evolutionarily conserved function in establishing the temporospatial expression of *Shh* in the MDO in vertebrates. Furthermore, Shh mediates Pax6 function in regulating the partition of the p2 domain into the epithalamus and thalamus.

## Background

The habenula, which is a paired midline structure residing at the posterior and dorsal surface of the thalamus, is present in virtually all vertebrates. The habenula receives inputs from the forebrain limbic system and the basal ganglia through the stria medullaris and projects to the monoaminergic nuclei in the midbrain and hindbrain via the fasciculus retroflexus (FR) [1,2]. The neural connections of the habenula imply that it plays an important role in modulating emotion, motivation and reward values. Indeed, dysfunction of the habenula has been implicated in psychiatric disorders, such as depression, schizophrenia and drug-induced psychosis [2,3]. Despite the importance of the habenula, relatively little is known about the molecular control of the specification and differentiation of this structure in vertebrates.

Based on gene expression patterns and morphological landmarks, the caudal forebrain is divided into three segments, prosomeres (p)1, 2 and 3 [4,5]. The p1 and p3 domains generate the pretectum and prethalamus, respectively, while the alar plate of p2 produces the epithalamus and the thalamus. The epithalamus gives rise to the habenula, together with the pineal gland and choroid plexus. Using genetic fate mapping, we have recently shown that thalamic neuron precursor cells express homeobox gene *Gbx2,* and the *Gbx2* lineage defines a sharp compartment boundary between the habenula and thalamus in the mouse embryo [6], demonstrating a clear segregation of habenular and thalamic neurons. In parallel, we have demonstrated that the segregation between p1 and p2 cells is regulated by the cell adhesion factor protocadherin 10b in zebrafish [7]. However, how the epithalamus diverges from the thalamus and pretectum is largely unknown.

Sonic hedgehog (Shh) is expressed in the ventral midline of the neural tube and functions as a morphogen to control the dorsoventral patterning of the entire central nervous system [8,9]. Uniquely, *Shh* is expressed in a transverse band at the border between p2 and p3, called the mid-diencephalic organizer (MDO) located at the prospective zona limitans intrathalamica (ZLI) [10,11]. Therefore, the developing diencephalon receives Shh signals from both dorsoventral and anteroposterior directions. Experiments in chicks and zebrafish have demonstrated that Shh is the key component of the MDO that controls development of the thalamus and the prethalamus in vertebrates [12-16]. Genetic studies in mice and fish have recently shown that Shh signaling regulates the division of thalamic progenitor regions into rostral (rTh) and caudal thalamic (cTh) domains [17-20]. Enhancing Shh activity shifts the border between rTh and cTh caudally, whereas decreasing Shh signaling shifts the border anteriorly [18,21,22]. Interestingly, cells in the caudal area of the thalamus seem refractory to alterations of Shh activity [18], and the effect of changed Shh function on epithalamic development has not been characterized in detail in the previous studies.

*Pax6,* which encodes a transcription factor containing a paired domain and a homeodomain, is broadly expressed in the developing forebrain of vertebrates [23-25]. In the absence of *Pax6*, although the regionalization of the diencephalon is initially preserved, formation of the prosomere boundary and axonal projections from the thalamus are severely disrupted in mice [25-28]. The formation of the epithalamus has never been characterized in *Pax6*-deficient vertebrates. However, it has been reported that the pineal gland is missing or greatly reduced in people lacking a functional copy of *PAX6*[29,30], suggesting that Pax6 protein may play a crucial role in the development of the epithalamus including the habenula. Interestingly, *Shh* expression and perhaps its activity are enhanced at the MDO of *Pax6*-deficient mouse embryos at embryonic day ((E) 10.5) [26,28]. Antagonistic interactions between *Pax6* and *Shh* are known to play a crucial role in patterning the spinal cord and the telencephalon [8,31]. However, it remains to be explored how Pax6-Shh interaction regulates the development of the mid-diencephalon, particularly the habenula.

The goal of this study is to investigate the molecular control of habenula development. To this end, we first analyzed the expression patterns of molecules that define different populations of habenular cells. Using these newly discovered habenular markers, we examined the genetic interactions between *Pax6* and *Shh* in the control of habenular development. We demonstrate that *Pax6* regulates the temporospatial expression of *Shh* at the MDO. Furthermore, Shh signaling plays a crucial role in regulating the specification of the habenula. Our results provide novel information on the development of the habenula and the MDO.

## Results

### Characterization of progenitors and precursors of habenular neurons

**Figure 1:**
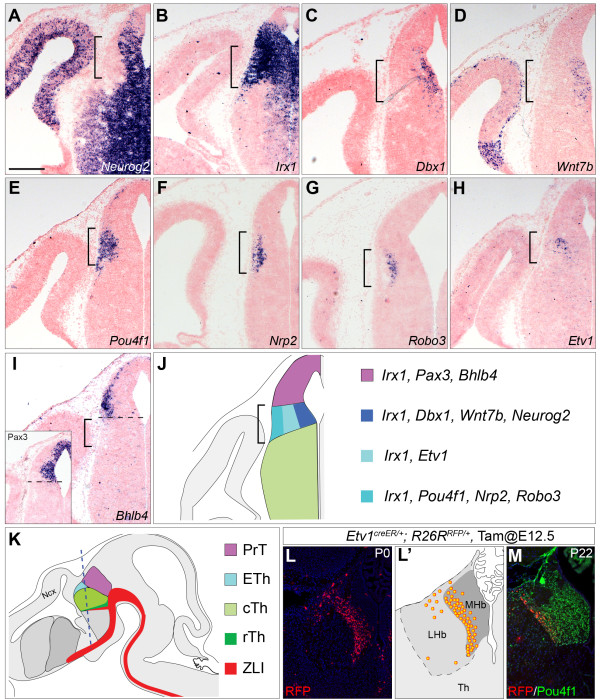
**Identification of molecular markers for the habenular precursor domain. (A-I)** *In situ* hybridization (ISH) for the indicated markers on coronal sections of wildtype embryos at embryonic day (E)12.5. The inset in I shows ISH for *Pax3*. Brackets indicate the presumptive habenular domain; the dashed line in I demarcates the border between the pretectum and epithalamus. **(J,K)** Schematic representation of coronal and sagittal sections of E12.5 brains, respectively. The dashed line in K indicates the plan of sections. **(L-M)** Contribution of descendants (RFP^+^) of *Etv1-*expressing cells that are labeled at E12.5 to the medial habenula (MHb) in *Etv1*^*+/creER*^*; R26R*^*+/RFP*^ at post-natal day (P)0 **(L)** or at P22 **(M)**. The distribution of the fate-mapped cells is summarized in L’ and the MHb is marked by Pou4f1 immunoreactivity **(M)**. cTh, caudal thalamus; ETh, epithalamus; LHb, lateral habenula; PrT, pretectum; rTh, rostral thalamus; ZLI, zona limitans intrathalamica. Scale bar in **(A)**: 200 μm **(A-I)**; 250 μm **(L)**; 196 μm **(M)**.

To investigate the developmental program that controls the subdivision of p2 into the epithalamus and the thalamus, we began by looking for molecular markers that define the epithalamus, particularly the habenula, in mouse embryos. We performed a detailed analysis of a variety of known developmental regulators that are differentially expressed in the diencephalon at E12.5, a stage when most habenular and thalamic neurons are being generated. As described previously [17], proneuronal gene *Neurog2* (previously named *Ngn2*) is broadly expressed in progenitors located in the thalamic ventricular zone (VZ) and also basal progenitors in the cTh (Figure 1A). *Neurog2* transcripts are also present in the VZ dorsal to the cTh, suggesting that *Neurog2* is also expressed in epithalamic progenitors (Figure 1A). *Irx1*, which encodes a protein belonging to the Iroquois homeodomain family, is expressed in the dorsal side of the diencephalon [32]. At E12.5, transcripts of *Irx1*, as well as those of paralogous genes *Irx2* and *Irx3*, are abundantly present throughout the epithalamus, pretectum and prethalamus, while a lower level of expression is seen in the VZ of the cTh (Figure 1B and data not shown). Homeobox gene *Dbx1* is initially expressed in p2 in a dorsal^high^-ventral^low^ gradient [18,33]. By E12.5, *Dbx1* transcripts are mainly restricted to the VZ of the presumptive epithalamus and are barely detectable in the cTh (Figure 1C). In addition to its strong expression in the prethalamus, a weaker expression domain of *Wnt7b* is present in the VZ of the epithalamus overlapping with that of *Dbx1* (Figure 1D). Collectively, our data suggest that the habenular progenitor domain is marked by the combinatorial expression of *Neurog2*, *Irx1*, *Dbx1* and *Wnt7b*.

We next analyzed genes that mark postmitotic habenular neurons. As described previously, *Pou4f1* (also known as *Brn3a*) is specifically expressed in the habenula [33] (Figure 1E). Axonal guidance genes *Robo3* and *Neuropilin 2* (*Nrp2*) are expressed in the habenula [33,34]. Transcripts of *Robo3* and *Nrp2* were detected in cells that emerged from the habenular progenitor domain marked by *Dbx1* and *Wnt7b* (Figure 1C-G). *Etv1*, which encodes a member of the ETS family of transcription factors, is expressed in the prospective habenula at E14.5 [33]. At E12.5, *Etv1* transcripts were detected in cells residing between the lateral-most area that was positive for *Pou4f1, Robo3* and *Nrp2* and the *Dbx1*^*+*^/*Wnt7b*^*+*^ VZ (Figure 1H). Although it has been suggested that *Bhlhe23* (previously called *Bhlhb4*) is expressed in the area caudal and dorsal to the thalamus including the epithalamus [35], *Bhlhe23* transcripts were mainly found in the lateral wall of the pretectum residing in an area dorsal and caudal to the *Pou4f1*+ domain (Figure 1E,I). Furthermore, the *Bhlhe23*^+^ domain was positioned parallel but more lateral to the expression domain of *Pax3*, which demarcates the pretectal VZ (Figure 1I and inset) [36,37].

To forge the link between the gene expression pattern in the embryo and the mature habenular nuclei after birth, we performed genetic fate mapping to examine the developmental fate of *Etv1*-expressing cells in the mouse embryo. We crossed the *Etv1*^*creER*^ knock-in mouse strain (JAX013048) and a cre-reporter line, *R26R*^*RFP*^, in which robust red fluorescence protein (RFP) will be permanently expressed upon cre-mediated excision of a STOP cassette flanked by two *loxP* sequences upstream of a cDNA encoding tdTomato RFP [38]. No RFP-labeled cells were detected in *Etv1*^*creER/+*^*; R26R*^*RFP/+*^ embryos that did not receive tamoxifen (data not shown), demonstrating that creER-mediated recombination is dependent on tamoxifen administration. To label *Etv1*-expressing cells, we administered tamoxifen to time-pregnant females carrying *Etv1*^*creER/+*^*; R26R*^*RFP/+*^ embryos, and examined the distribution of RFP-labeled cells at postnatal day (P) 0 and 22. In the caudal forebrain, descendants of *Etv1*-expressing cells labeled at E12.5 were mostly found in the medial habenular nuclei, which were delineated by Pou4f1 immunoreactivity, in *Etv1*^*creER/+*^*; R26R*^*RFP*^ mice at P0 and P22 (Figure 1L-M). Therefore, the *Etv1* expression domain in the diencephalon at E12.5 identifies precursors of the medial habenular nuclei.

In summary, we have identified several molecular markers that are expressed in distinct but overlapping domains of the prospective habenula at E12.5 (Figure 1J).

### Loss of Pax6 results in expansion of the pretectum and thalamus at the expense of the epithalamus

**Figure 2:**
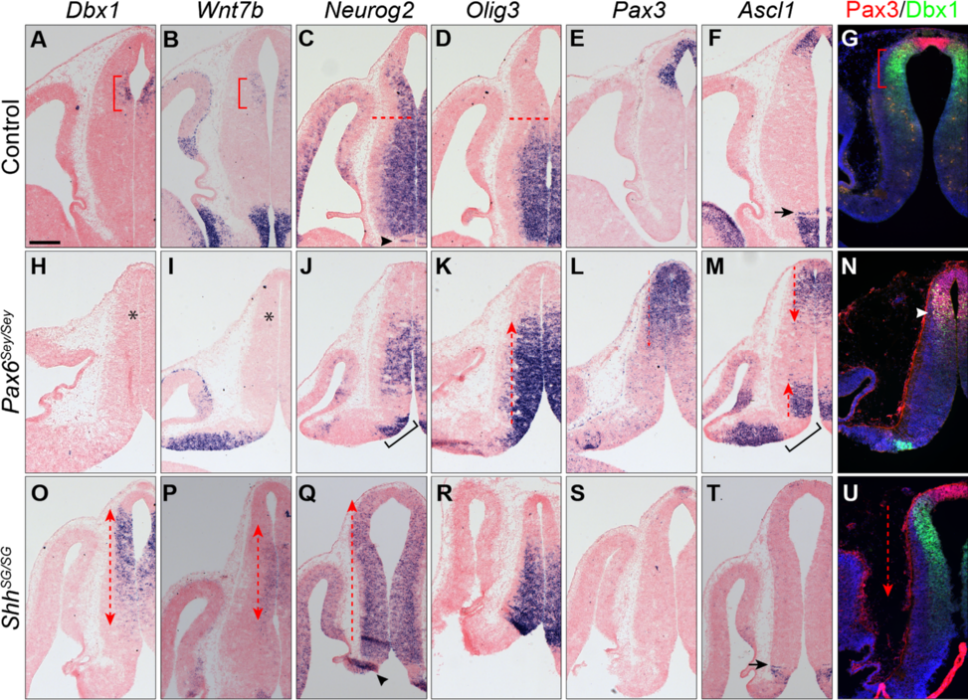
**Pattern formation of the diencephalon in embryos without Pax6 or with reduced Shh signaling. (A-F, H-M, and O-T)** ISH on adjacent coronal sections of E12.5 embryos. Probes and genotypes are indicated on the top and left of the panel. **(G, N, and U)** Immunofluorescence for Dbx1 and Pax3 on coronal sections of E12.5 embryos. Red dashed lines in **C** and **D** demarcate the border between the epithalamus and the thalamus; dashed line arrows indicate the expansion of the particular progenitor domain; black arrowheads and brackets in **C**, **J**, **Q** and **M** show the normal and the expanded ZLI, respectively; black arrows in **F** and **T** indicate the rTh domain; red brackets demarcate the prospective habenular domain; white arrowheads in **N** show cells positive for both Dbx1 and Pax3. Note that the apparent lack of transcripts of *Pax3* and *Asc1* in the pretectum in **S** and **T** is due to an anteriorly tilted sectioning plane. Scale bar in **(A)**: 200 μm **(A-F, H-M, and O-T)**; 241 μm **(G, N, and U)**. E, embryonic day; ISH, *in situ* hybridization; rTh, rostral thalamic; ZLI, zona limitans intrathalamica.

**Figure 3:**
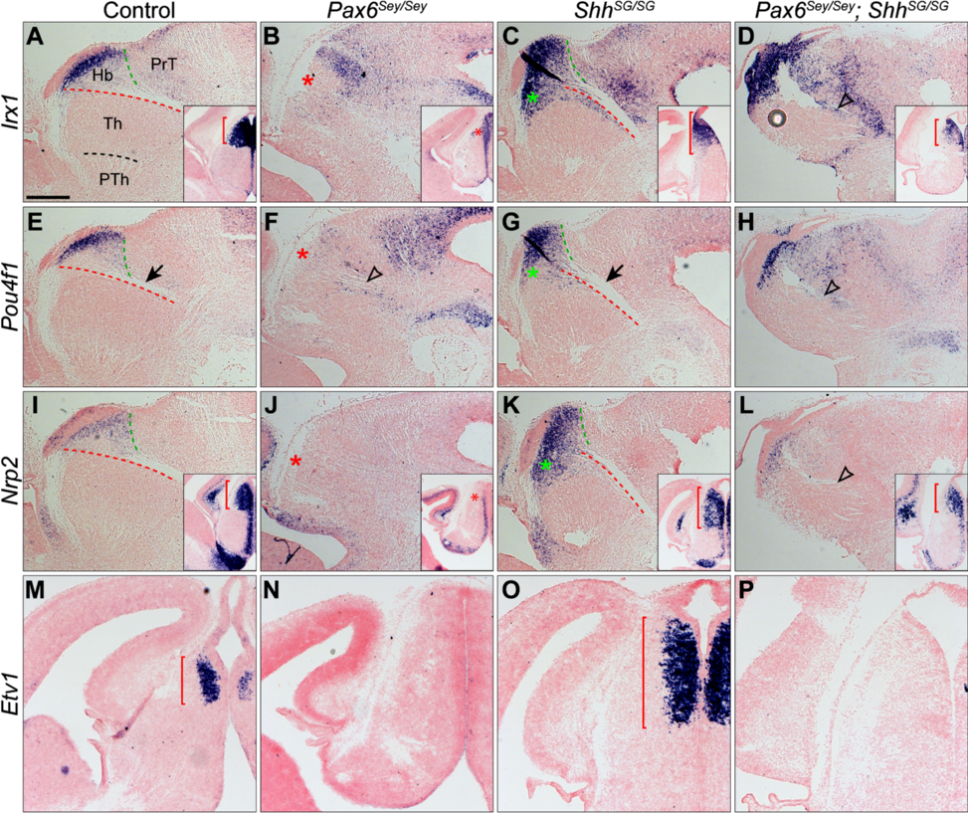
**Opposing function of *Shh *and *Pax6 *in regulating formation of the habenula. (A-P)** ISH for *Irx1, Pou4f1, Nrp2* and *Etv1* on sagittal **(A-L)** and coronal **(M-P)** sections of E14.5 embryos of indicated genotypes. Insets show coronal sections of E14.5 embryos. Red dashed lines indicate the posterior border of the thalamus demarcated by the FR tract (arrow); green dashed lines demarcate the posterior limit of the habenula; red brackets show the habenular nuclei; red and green asterisks indicate the loss and ectopic expression of habenular markers, respectively; empty arrowheads show abnormal defasciculating axonal fibers replacing the FR tract. Scale bar in **(A)**: 200 μm **(A-L)**; 170 μm **(M-P)**; 711 μm (insets). E, embryonic day; FR, fasciculus retroflexus; ISH, *in situ* hybridization.

To investigate if *Pax6* is essential for habenular development, we characterized the diencephalic phenotype in mouse embryos homozygous for a spontaneous *Small eye* (*Sey)* mutation, which is a null mutation in the *Pax6* gene [39]. The transcripts of *Dbx1* and *Wnt7b* were completely missing in p2 of *Pax6*^*Sey/Sey*^ embryos at E12.5 (Figure 2H,I). Furthermore, *Neurog2* expression in the presumptive habenular progenitor domain was also absent (Figure 2J). In concurrence with the abnormal specification of the habenular progenitor domain, transcripts of *Irx1*, *Pou4f1*, *Nrp2* and *Etv1* were mostly absent in the prospective habenular precursors of *Pax6*^*Sey/Sey*^ embryos at E14.5 (Figure 3B,F,J,N). These observations indicate that loss of *Pax6* disrupts the specification and differentiation of the habenula.

We next examined how loss of *Pax6* altered the patterning of the diencephalon, particularly the formation of the thalamus and the pretectum, as they reside in the ventral and caudal sides of the habenula, respectively. *Olig3* is normally expressed in the thalamus (rTh and cTh) and the ZLI [17] (Figure 2D). In *Pax6*^*Sey/Sey*^ embryos at E12.5, *Olig3* expression was maintained and its expression domain appeared expanded dorsally (Figure 2K). Because of the flexure of the neural tube, the pretectum, which is demarcated by the expression of *Pax3* and *Ascl1*, appeared dorsal to the epithalamus on a coronal section (Figure 2E,F). Immunofluorescence for Dbx1 and Pax3 showed that these two molecules are expressed in two juxtaposed domains, corresponding to the habenula and the pretectum, respectively, at E12.5 (Figure 2G). In *Pax6*^*Sey/Sey*^ embryos, the expression domains of *Pax3* and *Ascl1* were expanded anteriorly/ventrally so that the *Ascl1* and *Pax3* expression domain abnormally opposed or partially overlapped with that of the *Olig3*^+^ domain at E12.5 (Figure 2K-M). Remarkably, probably because of the protein perdurance, Dbx1 protein was still detectable in the presumptive habenula of *Pax6*^*Sey/Sey*^ embryos despite the lack of *Dbx1* transcripts by E12.5 (Figure 2H,N). However, many of these Dbx1^+^ cells abnormally expressed Pax3 (Figure 2N). This suggests that some habenular progenitors may be initially formed but adopt a pretectal fate in the absence of *Pax6*. Collectively, our data suggest that the thalamic and pretectal progenitor domains are respectively expanded dorsally and anteriorly at the expense of the epithalamus in the absence of *Pax6*.

**Figure 4:**
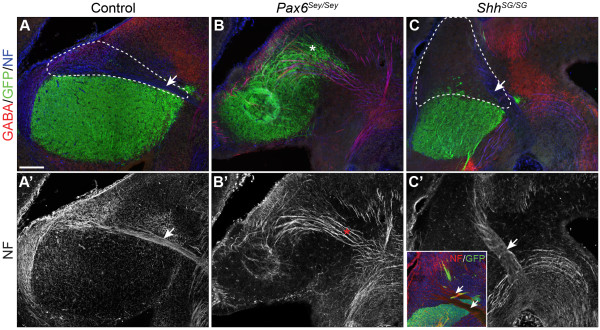
**The partition of p2 to form the habenula and the thalamus is controlled by the interaction between *Pax6 *and *Shh*. (A–C)** Immunofluorescence for GABA, GFP, and neurofilament (NF) on sagittal sections of E14.5 embryos of indicated genotypes. **(A’-C’)** the blue channel of images A-C to show for NF staining. The dashed line outlines the habenula (Hb); arrows indicate the FR tract; the asterisk shows defasciculating axonal fibers in place of the FR tract. Note that the FR tract becomes enlarged and supernumerary in *Shh*^*SG/SG*^ embryos (see inset in **C’**). Scale bar in **(A)**: 200 μm **(A-C’)**. E, embryonic day; FR, fasciculus retroflexus; GABA, gamma-aminobutyric acid; GFP, green fluorescent protein.

To confirm the abnormal expansion of thalamic and pretectal domains in *Pax6*^*Sey/Sey*^ embryos, we analyzed markers for thalamic and pretectal neurons. Homeobox gene *Gbx2* is broadly expressed in postmitotic thalamic neurons and the *Gbx2*-lineage exclusively contributes to cTh-derived nuclei [6,40]. Taking advantage of enhanced green fluorescence protein (EGFP) expression from the *Gbx2*^*creER*^ locus, which contains a *creER-ires-EGFP* cassette in the 5′-untranslated region (UTR) of the *Gbx2* gene [6], we examined the distribution of thalamic neurons in *Pax6*^*Sey/Sey*^ embryos at E14.5. Double labeling of GFP and neurofilament (NF) showed that the posterior border of the GFP^+^ thalamus was marked by the NF^+^ FR tract, which is formed by the habenular efferents [41] (Figure 4A,A’). Pretectal neurons are mostly gamma-aminobutyric acid (GABA)ergic and extend axonal fibers to the dorsal midline forming the posterior commissure (Figure 4A). In *Pax6*^*Sey/Sey*^ embryos at E14.5, GFP^+^ cells encroached the presumptive habenula and were intermingled with GABA^+^ neurons (Figure 4B,B’). The FR tract was replaced by defasciculating axonal fibers running from the GFP^+^ domain to the ventral midbrain (Figure 4B,B’). Interestingly, these axonal fibers were mostly positive for GABA, resembling posterior commissural axons rather than the FR tract (Figure 4B). Collectively, these data suggest that thalamic and pretectal neurons occupy the presumptive habenula in *Pax6*^*Sey/Sey*^ mutants.

In summary, our findings demonstrate that *Pax6* is essential for the development of the habenula. In the absence of *Pax6*, the habenula is lost in association with the dorsal expansion of the thalamus and anterior expansion of the pretectum.

### Pax6 is required for the establishment of the anlage of the MDO

**Figure 5:**
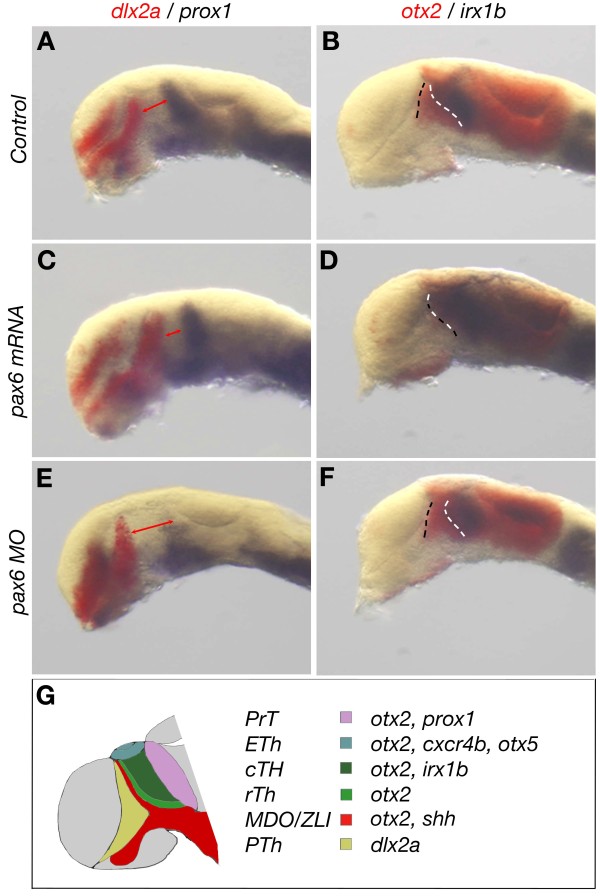
**Pax6 is essential for the establishment of the mid-diencephalon in zebrafish. (A-F)** Whole mount double *in situ* hybridization (ISH) for indicated markers of 28 hours post-fertilization (hpf) zebrafish embryos of indicated experimental procedure. Double red arrows mark MDO thalamic primordial. In **B**, **D** and **F**, black dashed line marks the border between MDO and the prethalamus, and white dashed line marks the border between MDO and thalamus. **(G)** Schematic representation of a sagittal section of zebrafish forebrain at 28 hpf. cTh, caudal thalamus; ETh, epithalamus; MDO, mid-diencephalic organizer; PrT, pretectum; PTh, prethalamus; rTh, rostral thalamus; ZLI, zona limitans intrathalamica.

We next investigated if Pax6 has a conserved function within the vertebrate lineage. Therefore, we analyzed Pax6 function during mid-diencephalic development in zebrafish. First, we analyzed the expression pattern of a prethalamic marker, *dlx2a,* and pretectal marker, *prox1*[16]. We found that both expression domains expand towards the MDO/thalamus anlage and simultaneously the MDO/thalamus primordia shrink in embryos overexpressed with *Pax6* mRNA (n = 7/11; Figure 5A,C,G). To block Pax6 function in zebrafish, we performed a morpholino oligomer-based double knock-down approach for both Pax6 homologs, Pax6a and Pax6b, which are expressed early in the diencephalon of zebrafish embryos [42]. Consistently, knock-down of Pax6 leads to a broadening of the MDO/thalamus anlage (n = 6/10) and in a few cases the pretectal expression of *prox1* becomes down-regulated (n = 3/10; Figure 5E). Next, we addressed the expression of *otx2,* a marker of the primordium of the MDO/thalamus, and *irx1b,* which is expressed in the thalamus (Figure 5B,G). We found that *irx1b* shifts anteriorly into the territory of the MDO anlage in embryos in which *pax6* mRNA is overexpressed (n = 8/13; Figure 5D), suggesting that Pax6 represses the MDO primordium. Consistently, knock-down of Pax6 function leads to a broadening of the MDO primordium, whereas the thalamus seems to be less affected (n = 6/13; Figure 5F). This suggests that Pax6 restricts the anteroposterior width of the MDO primordium in zebrafish.

### Pax6 is essential for regulating the expression of Shh in the prospective MDO

**Figure 6:**
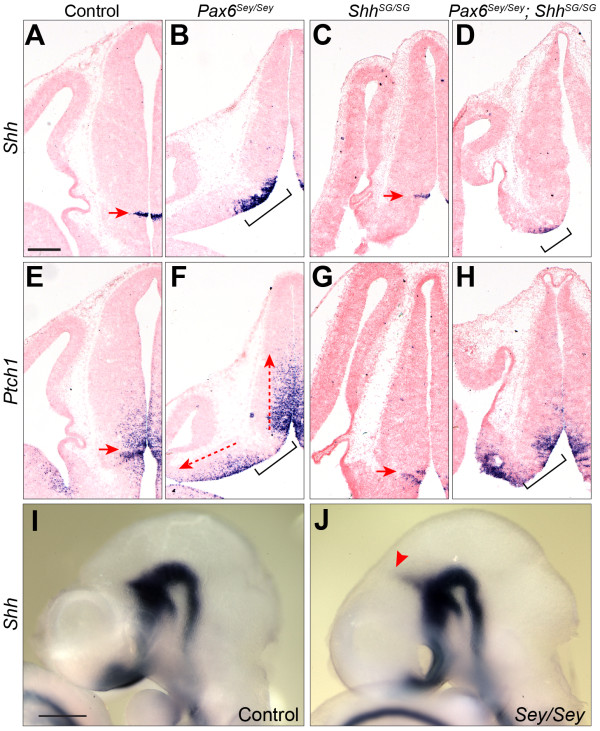
**Loss of *Pax6 *results in precocious formation and expanded expression of *Shh *in the MDO. (A-H)** ISH for *Shh* and *Ptch1* on coronal sections of E12.5 embryos of the indicated genotypes. **(I and J)** Wholemount ISH for *Shh* in control **(I)** and *Pax6*^*Sey/Sey*^**(J)** embryos at E9.5. Arrows denote the MDO; brackets show the expanded domain of *Shh* in the MDO; dashed line arrow indicates the dorsally (rostrally) and ventrally (caudally) expanded *Ptch1* expression domains in F; the red arrowhead in J indicates the precocious induction of the ZLI *Shh* expression in *Pax6*^*Sey/Sey*^ embryos. Scale bar in **(A)**: 200 μm **(A-H)**. E, embryonic day; ISH, *in situ* hybridization; MDO, mid-diencephalic organizer; ZLI, zona limitans intrathalamica.

*Pax6* is broadly expressed in the diencephalon before E10.5 and maintained in the habenula and prethalamus at E12.5 [23]. Previous studies have shown that loss of *Pax6* leads to expanded expression of *Shh* in the MDO [26,28], suggesting that *Pax6* plays an important role in regulating the MDO *Shh* expression. We confirmed that the *Shh* expression domain in the MDO was indeed expanded in *Pax6*^*Sey/Sey*^ embryos at E12.5 (Figure 6A,B). Furthermore, the expression domain of *Ptch1,* which is considered as a readout gene of Shh activity [43], was also expanded in both the thalamus and prethalamus in *Pax6*^*Sey/Sey*^ embryos at E12.5 (Figure 6E,F), demonstrating enhanced Shh signaling in the diencephalon lacking *Pax6*. It has been shown that activating Shh by forced expression of a constitutively active Shh receptor Smoothened (Smo) throughout the neural tube causes expansion of the rTh at the expense of the cTh [18]. In accordance with these findings, we found that the ZLI and the rTh, which are demarcated by the adjacent *Ascl1*^*–*^*/Neurog2*^*+*^ and *Ascl1*^*+*^*/Neurog2*^*–*^ domains, respectively [18], were noticeably enlarged in *Pax6*^*sey/sey*^ embryos at E12.5 (Figure 2C,F,J,M). Altogether, these results demonstrate that the loss of *Pax6* results in an expanded *Shh* expression domain and enhanced Shh activity at the ZLI.

We next investigated whether *Pax6* was required for the establishment of *Shh* expression in the ZLI. *Shh* is broadly expressed in the basal plate of the future diencephalon as early as E8.5 [20]. A wedge-shaped *Shh*^+^ domain corresponding to the prospective ZLI was detected in wildtype embryos at E10.5 but not at E9.5 (Figure 6I and data not shown). By contrast, the MDO *Shh* expression domain was clearly visible in *Pax6*^*Sey/Sey*^ embryos by E9.5 (n = 3/3, Figure 6J), demonstrating that the loss of *Pax6* results in precocious induction of MDO *Shh* expression in mice.

### Loss of Shh activity leads to expansion of the epithalamus at the expense of the thalamus at E12.5

To investigate whether the loss of the habenula could be attributed to altered *Shh* function in *Pax6*^*Sey/Sey*^ embryos, we first sought to determine if *Shh* is essential for habenular development. There is decreased cell proliferation and increased cell death in the entire diencephalon in mouse embryos homozygous for a *Shh*-null mutation at E9.5 [44]. To investigate the patterning role of *Shh* without the complication of growth defects in the diencephalon lacking *Shh* before E10.5, we performed analyses in mice homozygous for a *Shh* knock-in allele (designated as *Shh*^*SG*^), which produces Shh::GFP fusion protein with reduced Shh activity [45]. In *Shh*^*SG/SG*^ embryos at E12.5, although *Shh* expression was unchanged in the basal plate and ZLI, the expression domain of *Ptch1* was noticeably reduced in the thalamus at E12.5 (Figure 6C,G), indicating attenuated Shh activity in the p2 area in *Shh*^*SG/SG*^ embryos. Remarkably, the expression domain of *Dbx1* and *Wnt7b* in the presumptive epithalamus was expanded ventrally and overlapped with that of *Neurog2* and *Olig3* at E12.5 (Figure 2O-R). At E14.5, the expression domain of *Irx1*, *Pou4f1*, *Nrp2* and *Etv1* was noticeably enlarged at the expense of the thalamus in *Shh*^*SG/SG*^ embryos (Figure 3C,G,K,O). Immunofluorescence for GFP and NF in *Gbx2*^*creER/+*^*; Shh*^*SG/SG*^ embryos at E15.5 revealed that the GFP^+^ thalamus was evidently reduced in size compared with that of the control (Figure 4C). Furthermore, the habenula was expanded ventrally, and noticeably enlarged and multiple FR tracts were often detected in *Shh*^*SG/SG*^ embryos (Figure 4C,C’ and the inset). Our results demonstrate that *Shh* plays an important role in positioning the border between the epithalamus and the thalamus. Reducing Shh activity leads to enlargement of the habenula at the expense of the thalamus.

### Reducing Shh signaling partially restores the formation of the habenula in Pax6^Sey/Sey^ embryos

To define the epistatic relationship between *Shh* and *Pax6* in regulating habenular development, we investigated whether reducing Shh activity could rescue the habenula in embryos lacking *Pax6* by generating *Pax6*^*Sey/Sey*^; *Shh*^*SG/SG*^ double mutant embryos. Similar to that found in *Pax6*^*Sey/Sey*^ embryos, the MDO *Shh* expression domain was expanded in *Pax6*^*Sey/Sey*^; *Shh*^*SG/SG*^ double mutant embryos at E12.5 (Figure 6D). However, the range and the level of *Ptch1* expression in the thalamus in *Pax6*^*Sey/Sey*^; *Shh*^*SG/SG*^ embryos were more similar to those found in wildtype than those in either *Pax6*^*Sey/Sey*^ or *Shh*^*SG/SG*^ single mutant embryos at E12.5 (Figure 6E-H). These observations suggest that attenuated Shh protein activity may partially offset an increased *Shh* transcription at the MDO leading to relatively normal Shh signaling in the diencephalon in *Pax6*^*Sey/Sey*^; *Shh*^*SG/SG*^ embryos. Significantly, the expression domain of *Irx1*, *Pouf41* and *Nrp2* was largely restored in the presumptive habenular region in *Pax6*^*Sey/Sey*^; *Shh*^*SG/SG*^ double mutant embryos at E14.5 (Figure 3D,H,L, n ≥3 for each probe). These results indicate that *Shh* acts downstream of *Pax6* in controlling the formation of the habenula. Despite the rescue of some habenular markers, *Etv1* expression and the FR tract were still absent in *Pax6*^*Sey/Sey*^; *Shh*^*SG/SG*^ embryos at E14.5 (Figure 3D,H,L,P). Therefore, in addition to its important role in restricting *Shh*, *Pax6* may have an additional role in directly regulating habenular formation.

### Pax6 limits Shh expression in the MDO independent of Irx1b function in zebrafish

**Figure 7:**
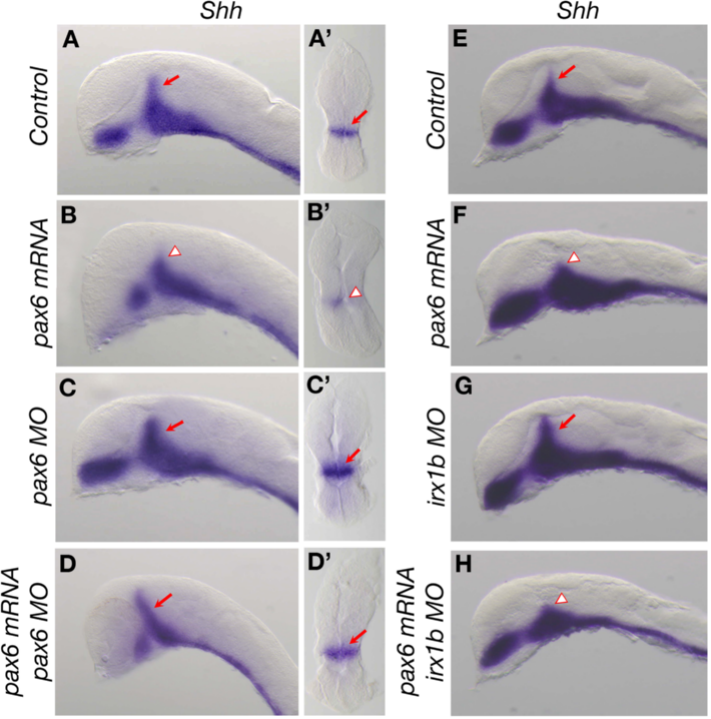
**Pax6 regulates the expression domain of *shh *expression in the MDO in zebrafish. (A-H)** Lateral views of wholemount ISH for *shh* in 28 hpf zebrafish embryos of the indicated experimental procedure. **(A-D)** red arrows mark the MDO, whereas red arrowheads mark the position of the reduced MDO. **(A’,B’,C’,D’)** show a coronal section of the prosencephalon with anterior to the bottom. hpf, hours post-fertilization; ISH, *in situ* hybridization; MDO, mid-diencephalic organizer.

Next, we investigated the role of Pax6 in the regulation of the MDO *Shh* expression in zebrafish. Overexpression of Pax6 led to a strong decrease of *shh* expression in the MDO in zebrafish (n = 16/26; Figure 7A-B’), consistent with the loss of Otx2 in the MDO primordium (Figure 5D). Similar to the loss of Pax6 in mice, blockage of the function of both zebrafish homologues *pax6a* and *pax6b* by morpholino oligomers led to an expansion of the MDO *shh* expression domain (n = 6/12; Figure 7C-C’); however, a precocious formation of the *shh* positive organizer was not observed in fish (data not shown). Co-injection of human *PAX6* mRNA restored the MDO *shh* expression in *pax6* double morphant zebrafish embryos (n = 3/7; Figure 7D,D’), demonstrating the specificity of the knock-down. In fish and mice, it has been shown that *irx1* controls the posterior limit of the MDO *shh* expression domain [15,46]. We found that overexpression of *PAX6* led to an anterior shift of *irx1b* expression into the primordium of the MDO (Figure 5D). Therefore, we asked whether the down-regulation of *shh* expression in Pax6-overexpressing embryos is mediated by *irx1b* or directly controlled by Pax6. Depletion of the *irx1b* function in zebrafish results in expanded *shh* expression in the MDO, similar to that found in *pax6* double morphant embryos (n = 6/9; Figure 7C,G). To determine the epistatic relationship between *pax6* and *irx1b* in regulating *shh* expression, we injected *pax6* mRNA in *irx1b*-MO embryos and found that the MDO *shh* expression domain was greatly reduced, similar to that found in *pax6*-overexpressing embryos (n = 4/8; Figure 7H). These data show that *pax6* acts downstream or in parallel with *irx1b* in restricting the MDO *shh* expression in fish.

Collectively, our results demonstrate that *Pax6* plays an evolutionarily conserved function in establishing the temporal and spatial expression domain of *Shh* in the prospective MDO organizer.

### Shh function from the MDO influences habenula formation

**Figure 8:**
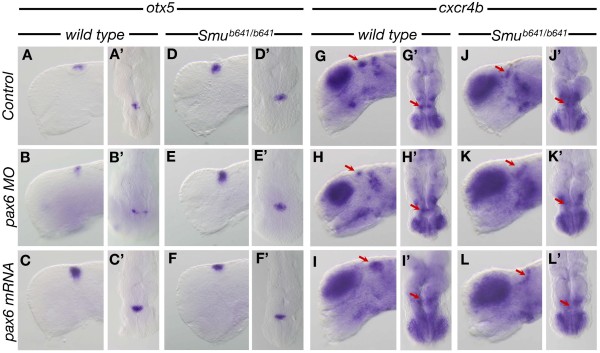
**Pax6 controls development of the epithalamus via regulation of MDO Shh function in zebrafish. (A-L’)** Lateral and dorsal views of wholemount ISH for *otx5***(A-F’)** and *cxcr4b***(G-L’)** in 28 hpf zebrafish embryos of the indicated experimental procedure. Red arrows marks the *cxcr4b* positive habenula precursor cells. hpf, hours post-fertilization; ISH, *in situ* hybridization; MDO, mid-diencephalic organizer.

In the next set of experiments, we investigated if Pax6 and Shh regulate the formation of the epithalamus in zebrafish in a way similar to that found in the mouse embryo. We used *otx5* as an early marker for the pineal gland, a key derivative of the epithalamus [47] and *cxcr4b* as a marker for habenula precursors [48]. We found that double knock-down of *pax6a/pax6b* led to a reduced expression of *otx5* (n = 7/15; Figure 8A-B’) and *cxcr4b* (n = 11/19; Figure 8G,H’). Conversely, overexpression of *pax6* mRNA led to an increase in the expression domain of *otx5* (n = 4/7; Figure 8C,C’) and *cxcr4b* (n = 6/10; Figure 8I,I’). This indicates that Pax6 positively regulates the size of the epithalamus in fish similar to that found in mouse embryos. We reasoned that the influence of Pax6 on epithalamus development could be mediated by Shh signaling from the MDO as found in the mouse embryo. Therefore, we analyzed epithalamus formation in embryos carrying a mutation in the Shh co-receptor smoothened (referred to as *Smu*^*b641/b641*^*mutants*) [49]. We found an increase in the epithalamic expression of *otx5* in *Smu*^*b641/b641*^ mutants (Figure 8D,D’ ,J,J’). Next, we blocked *Pax6* function in the *Smu*^*b641/b641*^ mutant background and found that in the absence of Shh signaling *Pax6* knock-down does not alter the size of the epithalamic *otx5* expression (Figure 8E,E’) and *cxcr4b* expression domain (Figure 8K,K’) compared to *Smu*^*b641/b641*^ mutant embryos (Figure 8D,D’ ,J,J’). Similarly, we found that overexpression of Pax6 in *Smu*^*b641/b641*^ did not change the epithalamus phenotype (Figure 8F,F’ ,L,L’) compared to *Smu*^*b641/b641*^ mutant fish. This suggests that Pax6 influences the development of the epithalamus - here the pineal complex and habenula - via regulation of Shh signaling at the ZLI organizer in the mid-diencephalon.

## Discussion

There is a surge of interest in the habenula because of its important role in cognitive and emotive behaviors [2,3,50,51]. However, the molecular underpinnings of the development of the habenula in vertebrates are still poorly understood. Expression profiling of postmitotic habenular neurons has recently demonstrated that the habenula represents a unique molecular territory in the central nervous system and is composed of heterogeneous cell types [33]. As a first step in understanding the specification and differentiation of habenular neurons, we have characterized the molecular markers that define habenular progenitors and precursors. Using genetic fate mapping, we have established a definite link between the medial habenular nuclei and *Etv1*-expressing progenitors in the epithalamus.

Transplantation and genetic manipulation experiments have demonstrated that Shh is the key molecule for MDO activity [12-14,16]. Experiments in the zebrafish have suggested that transcription factors, such *otx1/otx2*, *fezf2* and *irx1b*, form an interactive network to define the competent domain for the induction of *shh* in the prospective MDO [16]. This model, however, is not completely compatible with expression data and loss-of-function studies in mice [11]. The molecular mechanism underlying the formation of the MDO *Shh* expression in mammals remains to be determined. We found that loss of *Pax6* causes not only expanded *Shh* expression, but also precocious formation of the wedge-shaped *Shh* expressing domain in the prospective ZLI in mouse embryos by E9.5, at least 24 hours before the expression normally commences. To the best of our knowledge, this is the first report of accelerated formation of the MDO *Shh* expression domain caused by a mutation in vertebrates. It has been shown that Shh function in the ventral region is not essential for the induction of the MDO *Shh* expression [16,20]. In line with these conclusions, we found that the MDO *Shh* expression domain was similarly expanded in *Pax6*^*Sey/Sey*^ and *Pax6*^*Sey/Sey*^; *Shh*^*SG/SG*^ double mutants. These findings demonstrate that the temporospatial expression of *Shh* in the prospective ZLI is independent of Shh signals from the basal plate, but directly regulated by Pax6. Gain-of-function of *Pax6* results in reduced expression of the MDO *shh* expression in fish (Figures 5 and 7) and chick embryos as described recently [22]. Therefore, *Pax6* plays an evolutionarily conserved function in the establishment of the MDO *Shh* expression.

**Figure 9:**
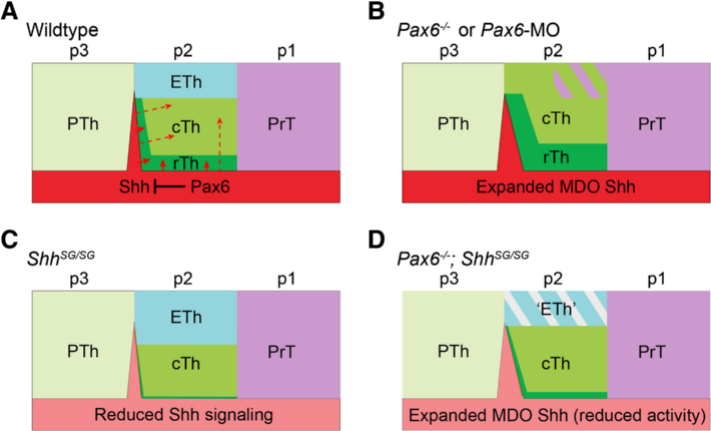
**Summary of subdivision of the diencephalon through interactions between *Shh *and *Pax6*. (A-D)** Schematic representations illustrate the partition of the diencephalon of indicated genotypes. In wildtype embryos **(A)**, *Pax6* restricts the expression domain of *Shh* in the MDO. Strong Shh signaling (arrows) induces rostral thalamus (rTh), while Shh appears to have a long-range effect (dashed line arrows) in inducing caudal thalamus (cTh) and inhibiting epithalamus (ETh). In *Pax6* loss-of-function mutants **(B)**, the MDO *Shh* expression domain is enlarged in mouse and zebrafish embryos, and the formation of the MDO *Shh* expression is accelerated in the mouse. The enhanced Shh activity causes expanded rTh and cTh and prethalamus (PrT) at the expense of the epithalamus. In embryos with reduced *Shh* function or gain of function of Pax6 **(C)**, the epithalamic domain is enlarged at the expense of cTh. In *Pax6* loss-of-function mutants with reduced Shh activity **(D)**, although the MDO *Shh* expression is enlarged, the epithalamus is partially restored. PTh, prethalamus; p1-3, prosomeres 1–3.

In thalamic explants, different concentrations of Shh proteins induced differential expression of rTh- and cTh-specific markers, suggesting that Shh acts as a morphogen to pattern p2 [12]. Indeed, increasing or reducing Shh activity alters the position of the border between rTh and cTh in relation to the ZLI [18,20]. Although the formation of the habenula was not explicitly characterized, altered expression of Dbx1 and Olig3, indicative of abnormal p2 subdivision, was reported in mouse embryos with excess or reduction of Shh activity [18]. By analyzing mouse embryos homozygous for *Sey* or a *Shh*-hypomorphic mutation and *Smu*^*b641/b641*^ mutant embryos or fish embryos knocked-down for Pax6 function, we showed that *Shh* plays a crucial role in positioning the border between the epithalamus and cTh, demonstrating that Shh has a long-range effect in the development of the entire p2. The far-reaching activity of Shh may be achieved by the combined action of Shh signals emanated from both the basal plate and the ZLI cells (Figure 9A). Future studies are required to determine if Shh signaling positions the border between the cTh and the habenula by directly inhibiting habenula fate or indirectly via promoting the cTh identity.

Antagonizing interactions between *Pax6* and *Shh* play an important role in dorsoventral patterning of the neural tube. In the spinal cord, loss of *Pax6* leads to expansion of the *Shh* expression domain in floor plate [31]. Furthermore, *Pax6* regulates the competence of the tissue in response to Shh signals [22,31]. Similarly, removal of *Pax6* partially rescues the medial ganglionic eminence marker in the forebrain of *Shh* mutants [8]. In the current study, we demonstrate a similar antagonistic interaction between *Pax6* and *Shh* in patterning the p2 alar plate. In the absence of *Pax6*, the development of habenula was blocked, while markers of the thalamus and pretectum were ectopically expressed in the presumptive habenula (Figures 2, 3 and 4 and summary in Figure 9B). Similarly, it has been shown that the expression domain of thalamic markers, *Vmat2* and *Ptn* (previously known as *Hbnf*), is shifted dorsally to the epithalamus in *Pax6*^*Sey/Sey*^ embryos [28]. Conversely, the expression of habenular markers was expanded into the thalamus in mouse and fish embryos with reduced Shh function or gain of function of Pax6 (Figures 2, 3, 8 and 9C). In addition, we observed partial co-expression of pretectal and caudal thalamic markers if Pax6 is inactivated, which suggests that Pax6 may also be involved in demarcating the cTh/pretectum boundary. Importantly, attenuation of Shh partially rescued expression of habenular markers in the presumptive epithalamus in *Pax6*^*Sey/Sey*^ embryos (Figures 3 and 9D). Furthermore, genetic experiments in zebrafish clearly demonstrated that *shh* acts downstream of *pax6* in controlling the formation of the pineal complex and habenular precursors (Figure 8). Collectively, our findings demonstrate that patterning defects in the p2 domain that is devoid of *Pax6* can, in part, be attributed to the precocious formation of MDO *Shh* expression and/or enhanced Shh activity in both mouse and fish embryos. It is worth noting that despite the rescue of some habenular markers, *Etv1* expression was missing and the FR tract was also absent in *Pax6*^*Sey/Sey*^; *Shh*^*SG/SG*^ embryos at E14.5 (Figure 4J-L and data not shown). It has been shown that *Etv1* is a direct transcriptional target of Pax6 in cortical development [52]. Furthermore, we found that deletion of *Etv1* resulted in abnormal formation of the medial habenular nuclei in mice (Guo and Li, unpublished observations). Therefore, in addition to its regulation of *Shh*, *Pax6* may also have a direct role in regulating the differentiation of the habenula.

## Conclusions

In this study, we have combined gene expression and genetic fate-mapping to define the progenitors of habenular nuclei. We demonstrate that *Pax6* is essential for establishing the temporospatial expression of *Shh* in the prospective ZLI in vertebrates. Furthermore, we demonstrate that Shh has a far-reaching effect in mediating *Pax6* function in controlling the p2 domain to diverge into the epithalamus, cTh, and rTh.

## Methods

### Maintenance of mouse and fish

All animal work has been conducted according to relevant national and international guidelines. Experimental procedures with mouse and fish were approved by the Animal Care Committee at the University of Connecticut Health Center and the Regierungspräsidium Karlsruhe (Aktenzeichen 35–9185.64) and the Karlsruhe Institute of Technology (KIT). All mouse strains were maintained on a mixed genetic background. Noon of the day on which a copulatory plug was detected was designated as E0.5. Mice carrying the *small eye* mutation *Pax6*^*Sey*^ were identified by the characteristic nasal and eye defects and confirmed with polymerase chain reaction (PCR) analysis as described previously [39]. Embryos carrying the *Gbx2*^*creER*^ and *Shh*^*GFP::Shh*^ alleles were identified by EGFP florescence in the spinal cord and by PCR genotyping as described previously [6,45]. At least three embryos of each genotype were examined and only the reproducible phenotype was described unless otherwise indicated.

Breeding zebrafish (*Danio rerio*) were maintained at 28°C on a 14 hour light /10 hour dark cycle. To prevent pigment formation, embryos were raised in 0.2 mM 1-phenyl-2-thiourea (PTU, Sigma, St. Louis, MO) after 24 hpf. The data we present in this study were acquired from analysis of KIT wild-type zebrafish AB_2_O_3_ as well as the *slow muscle omitted*^*b641*^ mutant line (referred to as *smu*) carrying a mutation in *smoothened homolog*[49].

### Functional analysis

Transient knock-down of gene expression in zebrafish was performed as described previously [7]. We used the following morpholino-antisense oligomers (Gene Tools, Philomath, OR) at a concentration of 0.3 mM each: *pax6a* MO (5′-TTTGTATCCTCGCTGAAGTTCTTCG-3′) and *pax6b* MO (5′-CTGAGCCCTTCCGAGCAAAACAGTG-3′) [53]. MO oligomers were injected into the yolk cell close to blastomeres at the one-cell or two-cell stage.

For mis-expression experiments full-length *pax6* and *irx1b* was cloned into a pCS2+ vector [15] and from this template mRNA was synthesized *in vitro* (Message Machine Kit, Ambion, Amersham, UK.). Together with rhodamine dextran (MiniRuby, Invitrogen, Carlsbad, CA) as lineage tracer, 350 pg mRNA per embryo was injected into the one-cell stage.

### Immunohistochemistry and *in situ* hybridization

Fish embryos were fixed in 4% paraformaldehyde/phosphate-buffered saline (PBS) at 4°C overnight for further analysis. Whole-mount mRNA ISHs were performed as described previously [54]. Embryonic mouse brains were dissected in cold PBS and fixed in 4% paraformaldehyde overnight for immunostaining or RNA ISH. Brains were cryoprotected and embedded in optimal cutting temperature (OCT) compound (TissueTek, Torrance, CA, USA) and sectioned in 20 μm thickness. Immunohistochemistry and ISH were performed as described previously [55]. Detailed protocols are available on the Li lab website [56]. The following antibodies were used in this study: rabbit anti-GFP (Invitrogen, Carlsbad, CA, USA); mouse anti-neurofilament, anti-Pax3, and anti-Pax6 (Developmental Study of Hybridoma Bank, University of Iowa, Iowa City, IA, USA); rabbit anti-GABA (Sigma); mouse anti-Pou4f1 (Santa Cruz, Biotechnology, Dallas, TX); rabbit anti-Dbx1, a gift from Y. Nakagawa (University of Minnesota, Minneapolis, MN, USA) [17]; Alexa secondary antibodies (Invitrogen). To compare the spatial expression domain of molecular markers, ISH and immunohistochemistry were performed on serial sections of the same embryo or carefully matched sections of different embryos.

## Competing interests

The authors declare that they have no competing interests.

## Authors’ contributions

SS and JL conceived and designed the study, and MC, QG and SW performed the experiments. All authors contributed to the data analysis. MC, SS and JL wrote the manuscript. All authors have read and approved the final manuscript.
